# Reversible doping and fine-tuning of the Dirac point position in the topological crystalline insulator Pb_1−*x*_Sn_*x*_Se *via* sputtering and annealing process†

**DOI:** 10.1039/d4na00821a

**Published:** 2025-02-10

**Authors:** Artem Odobesko, Johannes Jung, Andrzej Szczerbakow, Jędrzej Korczak, Tomasz Story, Matthias Bode

**Affiliations:** a Physikalisches Institut, Experimentelle Physik II, Universität Würzburg Am Hubland 97074 Würzburg Germany artem.odobesko@uni-wuerzburg.de; b Institute of Physics, Polish Academy of Sciences Aleja Lotników 32/46 02-668 Warsaw Poland; c International Research Centre MagTop, Institute of Physics, Polish Academy of Sciences Aleja Lotników 32/46 02-668 Warsaw Poland

## Abstract

In this study, we utilize scanning tunneling microscopy and spectroscopy to detail a sputter- and annealing methodology for preparing atomically clean Pb_1−*x*_Sn_*x*_Se(001) surfaces. We examine the impact these processes have on the surface quality, the composition, and the electronic properties. Our findings demonstrate that annealing temperatures between 250 °C and 280 °C produce smooth surfaces while maintaining the topological properties of Pb_1−*x*_Sn_*x*_Se. Fine control of the annealing temperature also allows for a reversible tuning of the doping level, enabling a positive or negative shift of the Dirac point energy with respect to the Fermi level. Our results highlight the effectiveness of these cleaning methods and demonstrate their potential for future research and applications in topological crystalline insulator materials.

## Abstract 2

We show that sputter-annealing a multicomponent topological material (Pb_1−*x*_Sn*_x_*Se) enables reversible tuning of the chemical potential while preserving its topological properties and achieving an atomically smooth surface.

## Graphical abstract

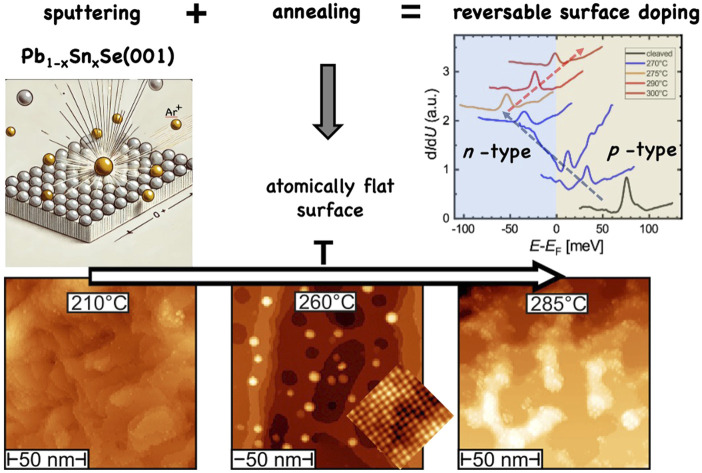

## Introduction

Topological crystalline insulators (TCIs) represent a novel class of materials where the nontrivial topology of electronic bands is protected by crystalline symmetry. Specifically, the IV–VI semiconductors SnTe, Pb_1−*x*_Sn_*x*_Te, and Pb_1−*x*_Sn_*x*_Se have been identified as TCIs due to their mirror symmetry with respect to the (110) plane. Unlike conventional topological insulators (TIs), the Dirac states in TCIs are protected by structural crystalline symmetries rather than time-reversal symmetry, allowing for unique electronic properties.^1,2^ In complex composite compounds like Pb_1−*x*_Sn_*x*_Se or Pb_1−*x*_Sn_*x*_Te, the topological phase is achieved by substituting Pb with Sn, leading to band inversion at certain compositions (*x* > 0.2) and temperature.^3,4^ This band inversion gives rise to topologically protected surface states, *e.g.*, the (001) surface exhibits two pairs of Dirac nodes located near the *X̄* and *Ȳ* points in the surface Brillouin zone. Gapless topological surface states were observed through angle-resolved photoemission spectroscopy (ARPES)^3,5–7^ and scanning tunneling microscopy (STM) and spectroscopy (STS).^8–10^

To prepare an atomically clean and structurally ordered surface, Pb_1−*x*_Sn_*x*_Se crystals are commonly cleaved along the (001) surface. Alternatively, epitaxially grown films can be prepared.^4,11,12^ However, achieving high-quality surfaces after exposure to ambient conditions is challenging. The desorption temperatures of native oxides PbO_*x*_ and SnO is high, which makes it difficult to clean surfaces from contaminations and native oxides by heating in ultra-high vacuum (UHV).^13,14^ The use of coating layers with passivating properties, such as Te^15^ or Se,^12^ can partially solve this task. Single-crystalline Te coatings effectively protect the surface, but oxidize upon prolonged air exposure and afterwards require high desorption temperatures, causing changes in stoichiometry, the crystalline structure, and the electronic properties of the sample. Se-based coatings require lower heating temperatures but are less practical due to their chemical volatility.

Previous studies have shown that surface cleaning of SnTe and PbTe can be achieved through ion bombardment and a combination of chemical etching and thermal annealing under UHV conditions.^16^ Treating SnTe surfaces with atomic hydrogen during the annealing process, effectively removes native oxides and carbon, enriching the surface with metals. However, the knowledge about surface quality and the electronic properties of the TCIs after post-treatment procedure as studied by STM and STS remains scarce.

Another issue often faced for topological materials is to control the position of the Fermi level. Although crystal growth in principle allows to control the doping, fine tuning the Fermi level is quite challenging,^17,18^ and the Dirac point is typically shifted relative to the Fermi level within a ≈±100 meV range only. A higher doping accuracy can be achieved by subsequent deposition of individual adatoms on the surface,^19^ however this process is irreversible and creates an additional high level of defects on the surface.

In this study, we prepare atomically clean and structurally ordered surfaces of Pb_1−*x*_Sn_*x*_Se (100) by Ar ion sputtering followed by annealing. The surface quality and electronic properties characteristic for TCIs are verified with STM and STS, respectively. We find that the annealing temperature is critical for obtaining atomically flat surfaces with large terraces, while preserving the topological states of the original surface. Moreover, our results reveal that thermal annealing affects the doping level of the sample. By controlling the annealing temperature, we consistently observe a reversible shift of the chemical potential from p-type to n-type and back to p-type, while maintaining the surface quality and topological properties of the sample. Our findings significantly advance the preparation procedures for atomically clean TCI surfaces for further research and applications.

## Methods

The experiments are performed in an UHV system equipped with an STM operated at a base temperature of *T* = 4.9 K. We study p-doped Pb_0.7_Sn_0.3_Se single crystals with initial Dirac point energies of 80 meV and 130 meV. Samples are cleaved at room temperature at a base pressure *p* < 10^−10^ mbar and immediately transferred into the STM. We use electro-chemically etched W tips, which are characterized on an Ag(111) surface to ensure their quality for STM and STS measurements. The differential tunneling conductance d*I*/d*U* is measured with lock-in technique with a modulation voltage *U*_mod_ = 2.5 mV at a frequency *f* = 790 Hz. STS measurements are performed at constant tip–sample distance.

Each Ar-ion sputtering cycle is carried out at a total pressure *p*_Ar_^+^ = 2 × 10^−6^ mbar with an ion energy *E*_Ar_^+^ = 350 eV. The sputtering rate is determined on Ag(111) to about ≈0.2 Ag monolayer (ML) per 1 min. We applied each sputtering step for a fixed duration of 15 min to consistently ensure the removal of 2–3 ML per cycle. To achieve more stable temperature conditions during multiple annealing cycles, samples are clamped between two metal blocks and secured with a screw to a flag-head holder, instead of the standard glueing procedure with silver epoxy.

## Results

Immediately after cleaving the pristine (001) surface of Pb_0.7_Sn_0.3_Se consists of flat terraces separated by step edges of varying heights. We are particularly interested in the step edges with a half-unit cell height because they break translational symmetry, resulting in a structural π-shift and the formation of a localized topological 1D edge mode, as demonstrated by Sessi.^20^ This edge mode displays intriguing topological behavior in magnetic fields,^21,22^ edge–edge hybridization,^23^ and interaction effects.^19^

**Fig. 1:**
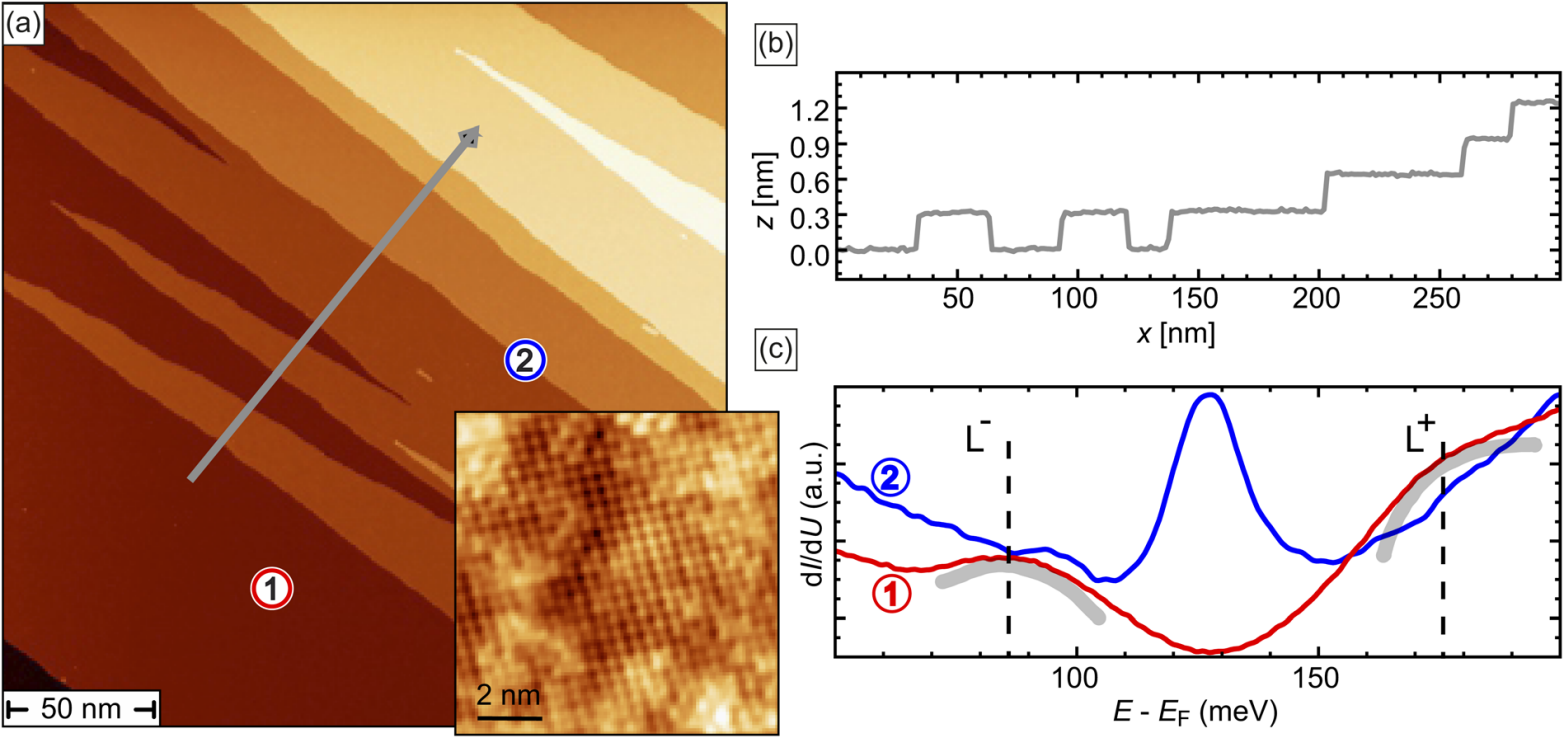
(a) Topographic STM image of a cleaved Pb_0.7_Sn_0.3_Se (001) surface with several steps of a height of a half-unit cell. The inset shows the atomically resolved Se sublattice. (b) Line profile measured along the black line in (a). (c) Typical tunneling spectra measured on a terrace (1) and at a single (2) step edge, exhibiting the characteristic van Hove singularity L^+,−^ and the edge mode at the Dirac energy *E*_D_ = (128 ± 5) meV. Stabilization parameters: (a) *U* = 200 mV, *I* = 10 pA, *I*_inset_ = 200 pA, (c) *U*_set_^1^ = 250 mV, *U*_set_^2^ = 200 mV, *I*_set_ = 200 pA.

Fig. 1(a) shows a typical STM image of a freshly cleaved (001) surface of Pb_0.7_Sn_0.3_Se. The surface comprises narrow terraces, which frequently form wedge-shaped protrusions. The step edges separating these terraces have a height of *h* ≈ 3 Å, as revealed by the *z*-profile shown in Fig. 1(b), measured along the gray arrow in Fig. 1(a). Since this height corresponds to half a unit cell, we anticipate observing topological 1D edge states at these step edges, as reported in ref. 20.

Two representative d*I*/d*U* spectra in are shown in Fig. 1(c). The red spectrum, marked ①, is measured on the flat terrace, whereas the blue spectrum, marked ②, is taken directly at the step edge. Spectrum ① exhibits a minimum at the Dirac energy, *E*_D_ ≈ 125 meV. Two symmetrically positioned shoulders, highlighted by gray curved lines, correspond to van Hove singularities associated with saddle points in the (001) surface electronic structure, as described in ref. 9, 10 and 24. Although these shoulders are broadened, their approximate positions are indicated with dashed lines labeled L^−^ and L^+^. In contrast, these features are less pronounced in the blue-line spectrum ②, which is typical for step edges with a half-unit cell height. Notably, spectrum ② exhibits a pronounced peak at the Dirac energy, signifying the presence of a 1D flat band characteristic of the topological edge state that emerges at half-integer step edges.

**Fig. 2:**
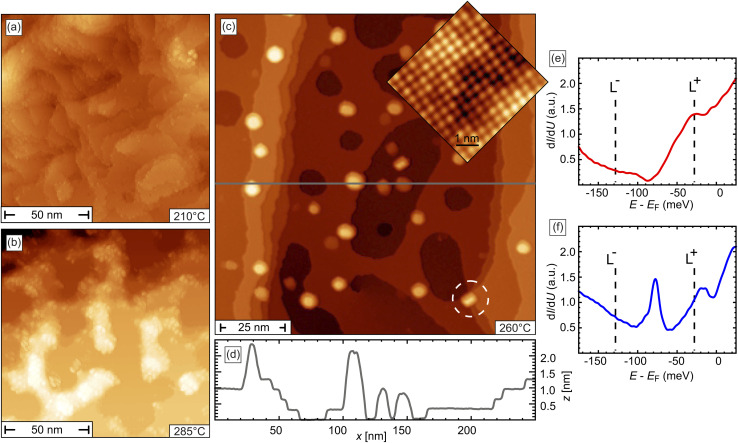
Topographic STM images of a (Pb,Sn)Se(001) surface after sputtering and subsequent annealing at (a) *T*_ann_ = 210 °C, (b) 285 °C, and (c) 260 °C. The inset in (c) reveals that atomic order is recovered after annealing at *T*_ann_ = 260 °C. (d) Line profile measured along the grey line in (c). (e and f) Single-point d*I*/d*U* spectra measured on a terrace and a half-unit cell step edge, respectively. The positions of the data points are shown in Fig. S5,† corresponding to the same surface as in (c), which was annealed at *T*_ann_ = 260 °C. Stabilization parameters: *U* = 1 V, *I* = 10 pA; (inset: *U* = 500 mV, *I* = 250 pA), *U*_set_ = 50 mV, *I*_set_ = 200 pA. Data are measured at *T* = 4.9 *K*.

To identify the optimal annealing parameters, we initially—after a single cycle of Ar^+^ sputtering of the freshly cleaved surface—systematically investigated the influence of a thermal treatment to the surface. Fig. 2(a)–(c) presents STM images of the surface after annealing for 25 min at (a) *T*_ann_ = 210 °C, (b) 285 °C, and (c) 260 °C, respectively. In Fig. 2(a) the topography is characterized by a large number of small terraces of varying sizes with irregular, rough edges. This observation indicates that up to an annealing temperature of at least 210 °C surface smoothing remains incomplete, for example because the thermal activation is insufficient to overcome the diffusion barriers and restore a smooth and well-ordered (Pb,Sn)Se surface. Thermal treatment at higher *T*_ann_ = 285 °C, see Fig. 2(b), results in a significantly degraded surface quality, as indicated by the roughness caused by large irregular protruding clusters which tend to accumulate at step edges. At an intermediate *T*_ann_ = 260 °C, see Fig. 2(c), we observe smooth areas of extended, atomically flat terraces and some small protruding clusters.

The data sets presented in Fig. 2(a)–(c) suggest that an annealing temperature of 260 °C is optimally suited to largely recover atomically smooth (Pb,Sn)Se upon Ar^+^ sputtering. In the following, we will analyze this surface in more detail to determine if the surface quality is comparable to pristine Pb_0.7_Sn_0.3_Se(001) as introduced in Fig. 1. Interestingly, the steps do not have a preferred orientation, unlike those in Fig. 1(a), where the steps are preferentially aligned along the cleaving direction. Instead, the edges appear rather rough or even curved. Yet, the line profile measured along the grey line in Fig. 2(c), which is presented in Fig. 2(d), confirms that the terraces are flat and separated by step edges of half-unit cell height (≈3 Å), in good accordance with the data presented in Fig. 1(a) and (b). This same line profile also crosses some clusters which have a typical diameter of about 10 nm. They can occasionally be found on the flat terraces, but are preferably located at single or bundles of step edges, as the one marked by a white circle in Fig. 2(c). The *z*-profile presented in Fig. 2(d) shows these clusters have a height of 1–2 nm.

The atomically resolved STM image in the inset of Fig. 2(c), taken on a flat terrace, reveals a well-ordered, defect-free (Pb,Sn)Se structure with a unit cell size of 4.2 Å, corresponding to the nearest anion–anion or cation–cation distance, derived from 
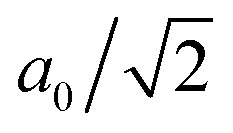
. The spectra measured on the terrace, shown in panel Fig. 2(e), clearly display one van Hove singularity (L^+^) and a minimum at the Dirac point. Furthermore, the spectrum measured at the position of a half unit-cell step edge exhibits a pronounced peak at the Dirac point, as shown in Fig. 2(f). Both spectroscopic features are hallmarks of the topological nature of the surface, indicating that the structural and electronic properties of sputter–annealed (Pb,Sn)Se are qualitatively comparable to those of freshly cleaved surfaces, as demonstrated in Fig. 1(c).

**Fig. 3:**
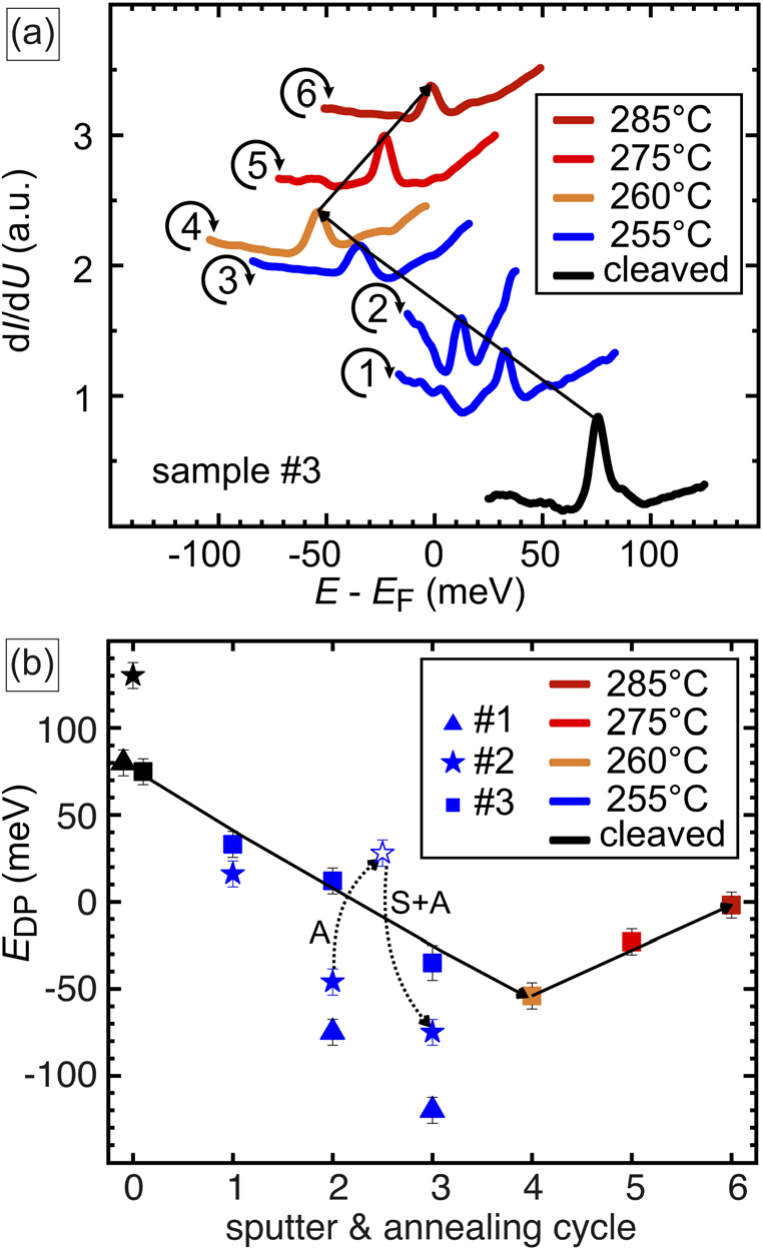
(a) Tunneling d*I*/d*U* spectra of the sample measured after cleaving (black curve) and following successive sputter–annealing cycles (cycles 1–6). The annealing temperatures for each cycle are color-coded as indicated in the legend. The spectra reveal that the position of the Dirac energy shifts to more negative or positive energies depending on the annealing temperature. (b) Dependence of the Dirac point energy, *E*_DP_, on the number of sputter–annealing cycles, as determined for three different samples (#1–#3).

Close inspection of the spectra reveals, however, that the position of the Dirac point significantly shifts upon application of the sputter–annealing sequence. While it is initially located at an energy *E*_DP_ = +128 ± 10 meV, it is found at *E*_DP_ = −85 ± 10 meV after the sputter–annealing cycle, indicating the transition from p-type to n-type doping. To further investigate this surface doping process in detail, we perform a systematic study of the electronic properties of half-unit cell step edges in dependence of the number of sputter–annealing cycles and the annealing temperature. The results are summarized in Fig. 3.

We examine three different samples in total. For the freshly cleaved samples, the DP is found at +80 meV in samples #1 and #3, while sample #2 exhibits a DP at +128 meV. Fig. 3(a) presents a representative series of spectra measured on sample #3. Immediately after cleaving, the LDOS peak is observed at +80 meV (black line).

The first three complete sputter–annealing cycles (1, 2, and 3) are performed under equal conditions, *i.e.*, annealing at 255 °C for 15 minutes, see blue lines in Fig. 3(a). The peak position gradually shifts towards lower energies with each cycle. After the third cycle, the DP crosses the Fermi level into the negative energy region, setting at approximately to −30 meV. This shift indicates a reversal of the surface doping from initially p-type to n-type after the third sputter-annealing cycle.

During the subsequent cycles (4, 5, and 6), the annealing temperature is gradually increased to 260 °C, 275 °C, and 285 °C, respectively. After cycle 4, the spectrum (orange line) shows further n-doping, with the DP shifting to approximately −50 meV. However, spectra from cycles 5 and 6 reveal that even a slight increase in annealing temperature, by just 15 °C, is sufficient to reverse the doping trend from n-type back towards p-type. For instance, after cycle 5 at *T*_ann_ = 275 °C (red line), the DP shifts positively to around −25 meV. Further increasing the annealing temperature to *T*_ann_ = 285 °C [dark red line, cycle 6 in Fig. 3(a)] results in the DP moving directly to the Fermi level.

The data obtained on all three samples #1, #2, and #3 are summarized in Fig. 3(b). The DP position is plotted along the *y*-axis, while the number of the sputter–annealing cycle is plotted along the *x*-axis. The annealing temperature for each cycle is color-coded, as shown in the legend. These three samples underwent different annealing treatments: sample #1, represented by triangles, was subjected to three identical sputter–annealing cycles (*T*_ann_ = 255 °C). It is evident the cycles results in an accumulating negative shift of the DP, with the total energy shift reaching Δ*E*_DP_ ≈ −200 meV. This shift represents the largest observed in this study and is comparable to the band gap of Pb_0.7_Sn_0.3_Se.

The data of sample #2, denoted by stars, is used to investigate the effect of annealing separately. For the freshly cleaved sample the Dirac point is energetically located at *E*_DP_ = +130 meV. After the first and the second sputter–annealing cycle, the DP shifts to +20 meV and to −50 meV, respectively. In the next step, sputtering is omitted, and the sample is only annealed for 15 minutes at the same *T*_ann_ = 255 °C. Consistent with the behavior observed for sample #3 in Fig. 3(a), the DP shifts towards more positive energies, reaching approximately +30 meV, as indicated by the dotted arrow labeled ‘A’ (annealing only). Reintroducing a full sputter-annealing cycle in the next step, again results in a n-doping with negative shift to *E*_DP_ ≈ −80 meV, see arrow labeled ‘S + A’ (for sputtering and annealing treatment). This highlights the importance of sputtering and suggests that defects accumulate near the surface, making it sensitive to sputtering over several monolayers.

Sample #3, represented by squares and discussed above for panel (a), is included to generalize the effects of different temperature treatments. It illustrates how the annealing temperature influences the sign of the surface doping effect, providing additional context to the observed trends.

It is worth noting that the surface quality gradually degrades after each cycle due to the increasing number of small accumulated clusters on the surface. This degradation continues until the surface becomes completely rough, typically after reaching 5–10 cycles.

## Discussion

The observed sputter- and annealing-induced reversal shift of the surface doping in Pb_1−*x*_Sn_*x*_Se can be attributed to several key factors: (i) presence of the intrinsic defects, (ii) changes in the stoichiometry and (iii) changes in chemical composition *x*.

In Pb_1−*x*_Sn_*x*_Se, intrinsic defects such as vacancies are crucial determinants of the material's doping behavior. Vacancies of Pb or Sn typically act as acceptors resulting in p-type doping. On the other hand, Se vacancies results in excess of electrons and leading to n-type doping. Additionally, interstitial defects—where atoms occupy non-standard lattice positions—can also influence the doping level. Thus the crystal is slightly deficient in Pb or Sn (leading to a higher concentration of vacancies), it is more likely to be p-type. Our atomically resolved images of the surface after several sputter–anneal cycles reveal no significant increase in the number of defects or vacancies on the surface itself. However, it is possible that these defects persist in the subsurface layers which cannot be entirely ruled out.

Stoichiometry refers to the ratio of cations (Pb and Sn) to anions (Se) in the crystal. Ideally, it should remain a 1 : 1 ratio, *i.e.* an equal number of cations and anions. This actual ratio directly influences the carrier concentration in the material. Even a small deviation in stoichiometry—around 0.1% of atomic composition—can shift the electrical conduction from n-type to p-type or *vice versa*. The observed doping effect can be attributed to the presence of native defects (including anti-sites) in the thin subsurface layer, where atoms substitute the lattice position for other elements. This means that subtle changes in the material's stoichiometry near the surface can significantly affect its doping level.

The chemical composition, denoted as *x*, refers to the proportion of Sn ions substituting for Pb in the Pb_1−*x*_Sn_*x*_Se compound. It quantifies the extent to which Sn replaces Pb in the crystal lattice. To observe changes in the electronic or structural properties induced by varying the chemical composition *x*, a substantial change in *x* is required—typically by several atomic percent. Minor adjustments in *x* are less likely to cause a noticeable shift in doping behavior compared to other factors, such as *e.g.* stoichiometry.

In contrast to ref. 7, where a transition from the topological to trivial phase was observed at slightly lower temperatures, the persistence of the topological edge state at step-edges with half-unit cell height in our experiments strongly suggests that any changes in the composition *x* are minimal and do not affect the topological phase of the material. Below *x* ≲ 0.2 at the studied temperatures, Pb_1−*x*_Sn_*x*_Se resides in a trivial phase where the topological edge state cannot exist.^4,20^ Consequently, for samples with *x* = 0.3 investigated here, any sputter- and annealing-induced changes in composition are expected to be minor and insufficient to trigger a transition of the material out of its topologically non-trivial phase.

Furthermore, Auger Electron Spectroscopy (AES) data of the surface reveal that while the general surface composition remains consistent after sputtering and annealing, the technique's resolution and sensitivity are insufficient to reliably detect subtle compositional changes (see Fig. S1 in ESI Material†). Consequently, within the error of our measurements, we cannot unambiguously determine minor stoichiometric variations or exclude their potential contribution to the observed doping behavior.

At the same time, both sputtering and annealing processes may potentially induce changes in the stoichiometry at the surface of the Pb_1−*x*_Sn_*x*_Se due to element-specific sputtering rates and different thermally activated mobilities. However, it was shown that the Ar^+^ sputter rates of Pb_1−*x*_Sn_*x*_Se alloys are independent of the detailed composition at Sn concentrations up to *x* < 0.56.^25^ Our data however unambiguously show, that thermal annealing without prior sputter cycle leads to the p-doping shift of the doping level of the crystal surface.

Thus, we can assume that the critical factor in the reversible tuning of the surface doping—and consequently the Dirac point position—lies in the introduction and subsequent healing of Se vacancies during the sputtering and annealing processes. Se, being the lightest and most chemically volatile element in the composition, is particularly susceptible to sputtering and thermal migration. The annealing process in a relatively narrow temperature window (250 °C ≲ *T*_annealing_ ≲ 280 °C), enables the controlled reintroduction of Se atoms to the surface, potentially through segregation from the bulk, and facilitates the healing of surface vacancies. The optimal annealing temperature is a compromise for *T* being low enough to avoid excessive evaporation of Se from the surface region but high enough to guaranty proper surface mobility of atoms needed for re-shaping of crystal surface.

In summary, we studied with the help of STM and STS an alternative method to prepare atomically clean and flat (Pb,Sn)Se surfaces through sputtering and annealing processes. This preparation circumvents the limitations of cleaving and allows for a fine-tuned control over surface quality and stoichiometry. Our findings demonstrate that annealing temperatures between 250 °C and 280 °C produce smooth surfaces while preserving the topological properties of Pb_1−*x*_Sn_*x*_Se. STS data also reveal that this preparation method allows for the reversible and precise tuning of the Dirac point position with respect to the Fermi level, enabling both n-type and p-type doping scenarios. Such control over the electronic properties opens new perspectives towards an *in situ* modification of the Dirac electron-mediated interactions and other quantum effects.

## Data availability

All data supporting the findings of this study are fully presented within the manuscript and its associated figures. No additional datasets were generated or analyzed for this article outside of those provided in the published content.

## Author contributions

Samples were grown by A. S., J. K., and T. S.; J. Y. and A. O. conceived the experiments; J. Y. conducted the measurements and analyzed the data; M. B. provided project management and funding acquisition; J. Y. and A. O. wrote the manuscript with input from all authors. All authors discussed the results.

## Conflicts of interest

The authors declare no competing interests.

## Supplementary Material

NA-OLF-D4NA00821A-s001
