# Supplementary material for: Reversible doping and fine-tuning of the Dirac point position in the topological crystalline insulator Pb1−xSnxSe via sputtering and annealing process
Source: Nanoscale Adv. 2025 Feb 10;7(7):1885–91. doi: 10.1039/d4na00821a (PMC11808273; doi:10.1039/d4na00821a)
Supplement: NA-007-D4NA00821A-s001 [file NA-007-D4NA00821A-s001.pdf]

Supplementary Information (SI) for Nanoscale Advances.
This journal is © The Royal Society of Chemistry 2025

— Supplementary Material —

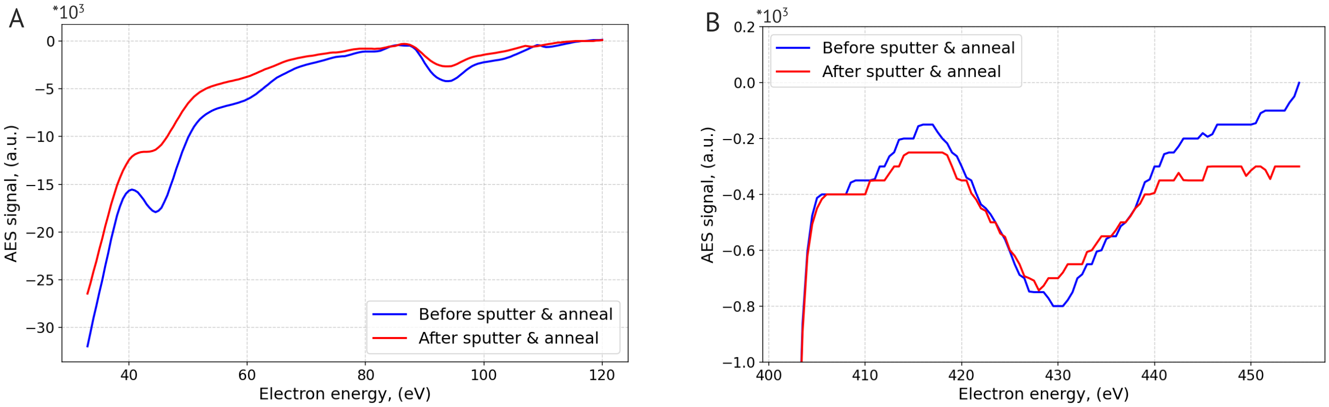

FIG. S1. AES spectra of $Pb_{1-x}Sn_xSe$ p-doped sample before and after the sputter and anneal cycle, shown in blue and red, respectively. (A) Spectra in the energy range (30–120) eV, highlighting the Auger peaks for Se at 47 eV and Pb at 97 eV. (B) Spectra in the energy range (400–460) eV, corresponding to the Sn Auger peak at 432 eV. Measurement parameters: $U_{\mathrm{mod}} = 10\,\mathrm{V}$, time constant $= 3\,\mathrm{s}$.

The AES spectra, measured on a freshly cleaved sample and after the sputter-anneal cycle, exhibit Auger peaks characteristic of the constituent elements: Se (atomic number 34) with peaks at 47 eV and 104 eV, Sn (atomic number 50) with a peak at 432 eV, and Pb (atomic number 82) with a peak at 97 eV.

A key challenge lies in quantifying the Se concentration. The Se Auger peak at 47 eV closely overlaps with the tail of the true secondary electron emission signal, complicating precise peak intensity identification. Additionally, the secondary electron emission coefficient is subtly influenced by the sample position, which changes during sample manipulation, such as the sputter-anneal process or STM measurements. These variations result in minor intensity shifts in the AES spectra (as can be seen in vertical shift of red and blue curves in Fig.S1), making the determination of small compositional changes in $Pb_{1-x}Sn_xSe$ ambiguous within experimental error.

To enhance the accuracy of compositional analysis, advanced electron detectors with higher resolution and improved energy filtering capabilities are essential. Such instrumentation would allow for better isolation of the Auger peaks from the background, enabling more reliable determination of subtle compositional deviations.

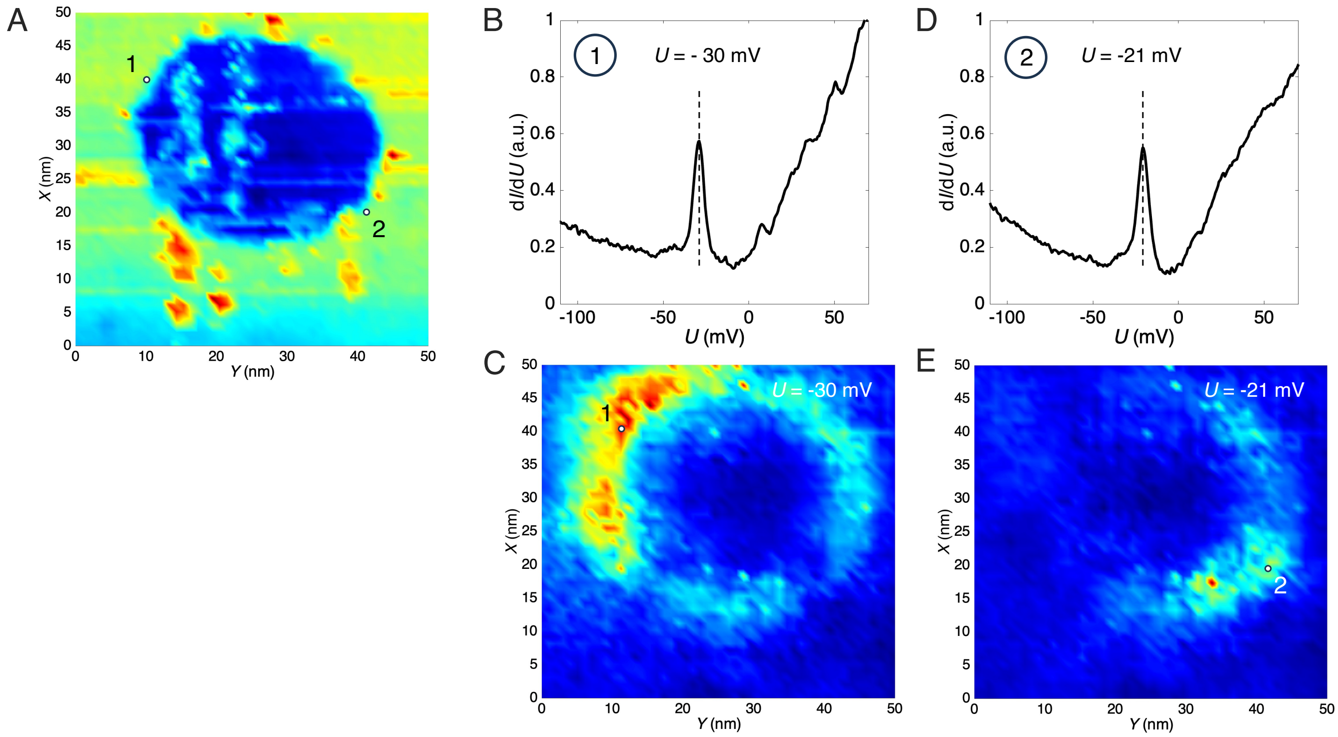

FIG. S2. Spatial variation of the Dirac point (DP) position along the step edge with a height of half a unit cell in the p-doped $Pb_{1-x}Sn_xSe$ sample after a sputter and anneal cycle. (A) Topography image showing a circular depression with a diameter of ≈ 30 nm and step height of half a unit cell (3 Å). (B) dI/dU point spectrum measured at the step edge at position 1, marked in A, where the DP is located at -30 mV. (C) Spatial map of the dI/dU signal measured at a tunneling voltage of $U = -30$ mV. (D) dI/dU point spectrum measured at the step edge at position 2, marked in A, where the DP is located at -21 mV. (E) Spatial map of the dI/dU signal measured at a tunneling voltage of $U = -21$ mV. Stabilization parameters: $U = -120$ mV, $I = 200$ pA, $U_{mod} = 1$ mV.

Figure S2 illustrates the spatial variation of the Dirac point (DP) position within a 50×50 nm area, showing a slight shift in the step-edge state energy position. As shown, the two opposite points marked as positions 1 and 2 in Fig. S2A exhibit different DP positions, shifted by approximately 10 mV. This behavior is also commonly observed in freshly cleaved samples, where DP fluctuations of ± 10 mV are typical.

In Fig. S2C, the dI/dU spatial map measured at $U = -30$ mV clearly shows a strong signal near position 1, corresponding to the DP at -30 mV. This signal fades away as the distance from position 1 increases and is almost negligible near position 2. Conversely, Fig. S2E, the dI/dU map at $U = -21$ mV, reveals a strong signal around position 2, which fades away from this point.

These observations suggest that the sputter and anneal process does not introduce significant additional fluctuations in the Fermi level due to inhomogeneities in the doping distribution, as compared to freshly cleaved samples. The variation in the DP position remains within the typical fluctuation range of ± 10 mV.

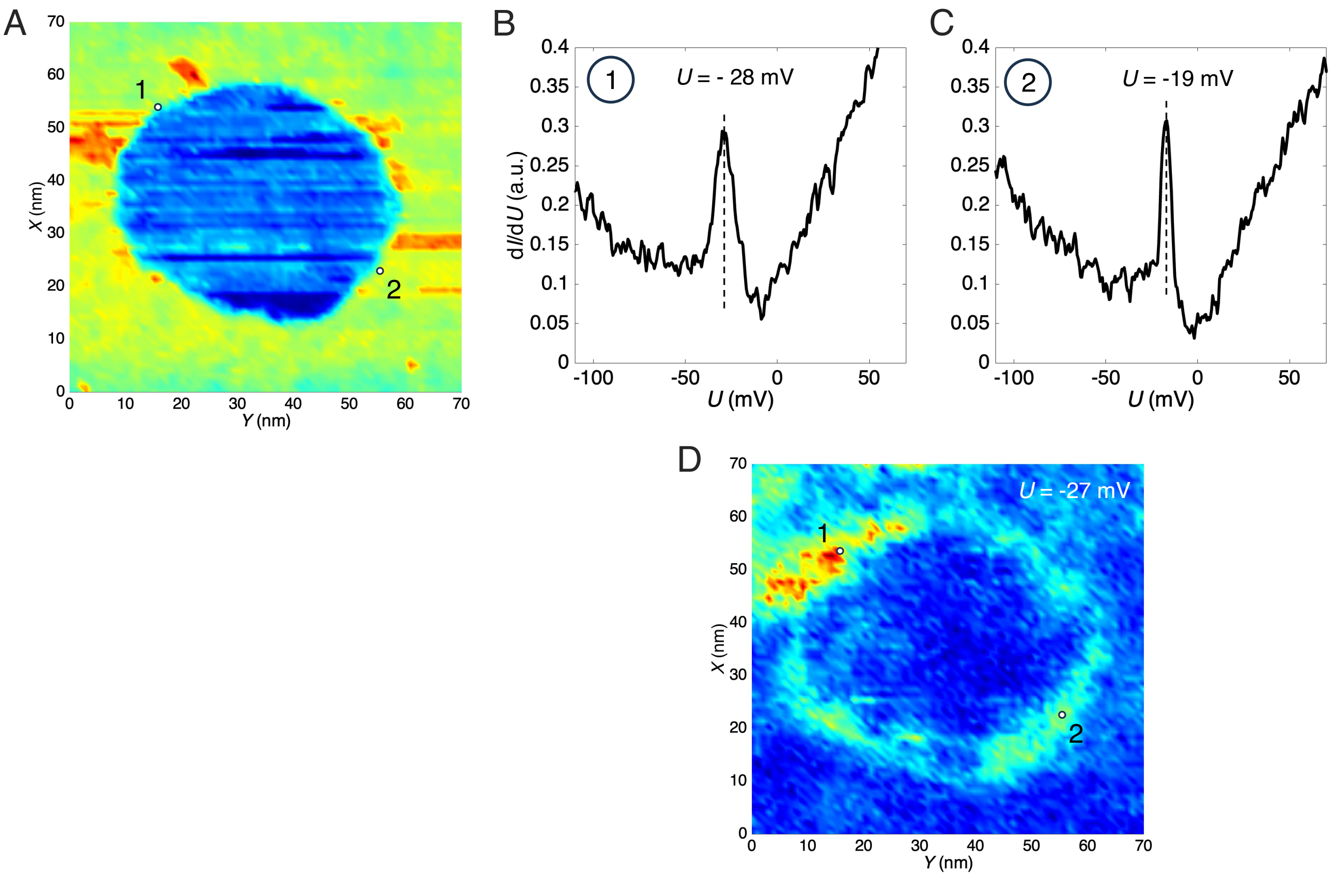

FIG. S3. Topological 1D edge state across the step edge with a height of half a unit cell in the p-doped $Pb_{1-x}Sn_xSe$ sample after a sputter and anneal cycle. (A) Topography image showing a circular depression with a diameter of ≈ 50 nm and a step height of half a unit cell (3 Å). (B) and (C) dI/dU point spectra measured at the step edge at positions 1 and 2, marked in A, with the Dirac point located at -28 mV and -19 mV, respectively. (D) Spatial map of the dI/dU signal measured at a tunneling voltage of $U = -27$ mV. Stabilization parameters: $U = -120\,\text{mV}$, $I = 200\,\text{pA}$, $U_{\text{mod}} = 1\,\text{mV}$.

Figure S3 illustrates the distribution of the 1D topological edge state along the step edge within a circular depression with a diameter of ≈ 50 nm. The edge state is observed along the entire perimeter of the step edge, confirming its presence throughout. As shown in Fig. S3B and Fig. S3C, the DP positions at the two measurement locations are shifted by approximately 10 mV, consistent with observations discussed in the previous section.

In Fig. S3D, the dI/dU spatial map measured at $U = -27$ mV highlights the presence of the edge state as regions of high-intensity signal distributed along the entire circular step edge. Variations in the signal intensity reflect slight deviations of the DP position from the tunneling bias voltage $U = -27$ mV at which the map was acquired. A weaker signal corresponds to a greater offset of the DP from the set bias.

The data demonstrate that the Fermi level shifts induced by the sputter and anneal cycle are uniform across the surface. Moreover, the edge state, attributed to the breaking of translational symmetry across the half-unit-cell step edge, is consistently observed at all locations along the step edge. This rules out the possibility that the enhanced signal observed in individual point spectra (Fig. 3 in the main text) originates from localized states caused by defects, such as impurities.

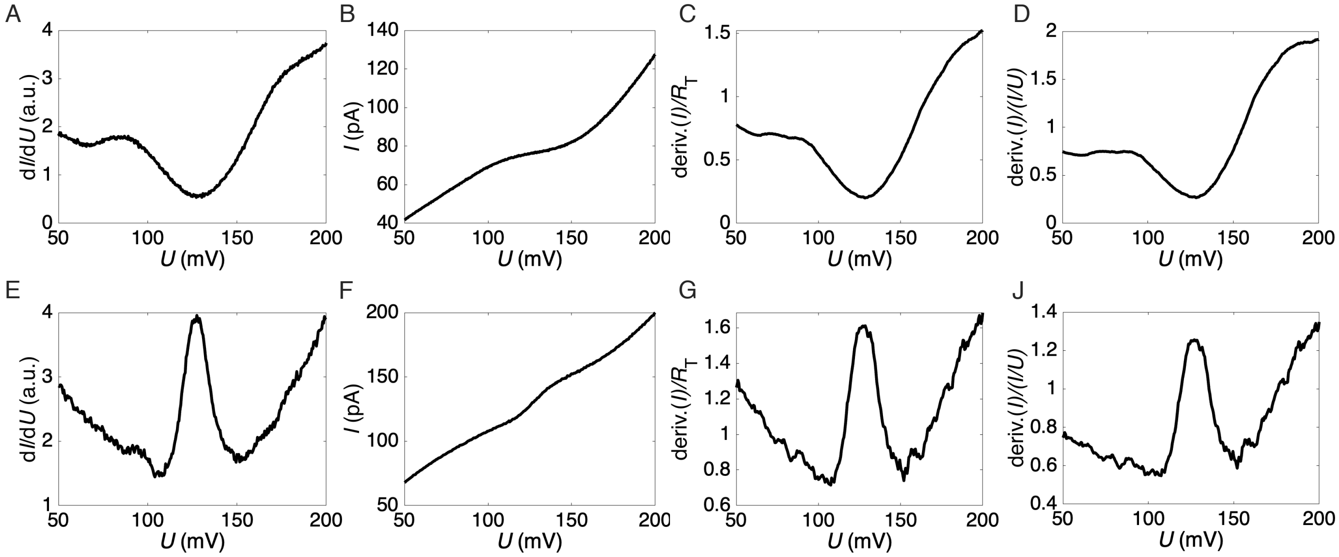

FIG. S4. Different representation of tunneling spectra measured on a terrace (upper row) and at a step edge (bottom row) presented in Fig.1(c). (A) and (E) dI/dU signal measured with Lock-In technique. (B) and (F) corresponding $I/(U)$ signal. (C) and (G) numerically calculated derivative of $I/(U)$ signal normalised by $R_{\mathrm{T}} = I_{\mathrm{set}}/U_{\mathrm{set}}$. (D) and (J) numerically calculated derivative of $I/(U)$ signal normalised by varying tunneling resitance $I/(U)$. Stabilization parameters: (A-D) $U_{\mathrm{set}} = 250$ mV, $I_{\mathrm{set}} = 200\,\mathrm{pA}$, $U_{\mathrm{mod}} = 1\,\mathrm{mV}$; (E-J) $U_{\mathrm{set}} = 200$ mV, $I_{\mathrm{set}} = 200\,\mathrm{pA}$, $U_{\mathrm{mod}} = 1\,\mathrm{mV}$.

Figure S4 presents tunneling spectra measured using the Lock-In technique, alongside simultaneously acquired I-V tunneling characteristics. The I-V data were further analyzed and processed to obtain the normalized, numerically calculated dI/dU, as shown in panels (C,D) and (G,J).

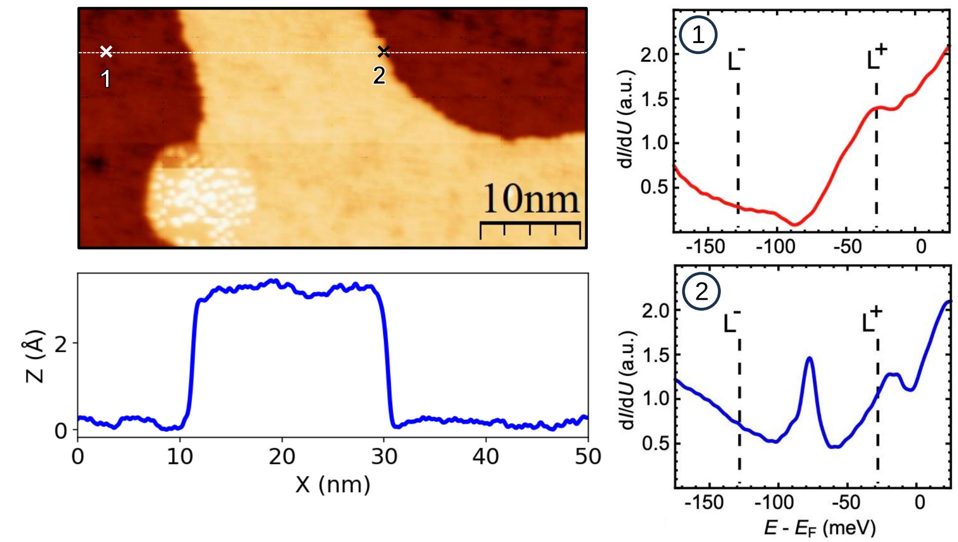

FIG. S5. STM image of the surface sputtered and annealed at $T_{\text{ann}} = 260°$C, similar to Fig.2(c). The line profile along the dashed line reveals a terrace with a half-unit cell height. Positions 1 and 2 indicate where the single-point dI/dU spectra shown in Fig.2(e, f) were measured—on a terrace and a half-unit cell step edge, respectively.

Topographic STM images of a $Pb_{1-x}Sn_xSe$ surface after sputtering and annealing at 260°C. The image was taken with an X, Y offset of 450 nm, 450 nm from the location shown in Fig.2(c). It marks the positions where the tunneling spectra dI/dU presented in Fig.2(e, f) were acquired.
